# Extracting antibiotic susceptibility from free-text microbiology reports using natural language processing

**DOI:** 10.1017/ice.2025.10210

**Published:** 2025-09

**Authors:** Andrew Chou, Ronald George Hauser, Lori A. Bastian, Cynthia A. Brandt, Barbara W. Trautner

**Affiliations:** 1 VA Connecticut Healthcare System, West Haven, CT, USA; 2 Section of Infectious Diseases, Department of Internal Medicine, Yale School of Medicine, New Haven, CT, USA; 3 Department of Biomedical Informatics and Data Science, Yale School of Medicine, New Haven, CT, USA; 4 Department of Laboratory Medicine, Yale School of Medicine, New Haven, CT, USA; 5 Department of Internal Medicine, Yale School of Medicine, New Haven, CT, USA; 6 Department of Emergency Medicine, Yale School of Medicine, New Haven, CT, USA; 7 Center for Innovations in Quality, Effectiveness and Safety (IQuESt), Michael E. DeBakey VA Medical Center, Houston, TX, USA; 8 Section of Health Services Research, Department of Medicine, Baylor College of Medicine, Houston, TX, USA

## Abstract

There is a clinical need to appropriately apply large language model (LLM)-based systems for use in infectious diseases. We sought to use LLM and machine learning for extracting antibiotic susceptibility from clinical microbiology free-text reports, allowing use for outbreak detection, increasing information gathering efficiency, and public health reporting.

## Introduction

Interest in artificial intelligence and machine learning (AI/ML) has rapidly gained broad attention, particularly since the November 2022 release of the ChatGPT large language model (LLM) chatbot. The LLMs have sparked the imagination of both the lay public and researchers while also generating immense interest into potential applications.^1^ In addition to chatbots, LLMs can be trained, or fine-tuned, for classification tasks. Despite the promise of LLMs,^2^ tangible use cases applying this new technology to electronic health record (EHR) data for clinical infectious disease uses are limited,^3^ and there is need for infectious disease experts’ participation and leadership to generate data needed to guide development and deployment of LLM-based systems for use in infectious diseases.^1^

We sought to evaluate and compare LLM and ML models for clinical infectious diseases information extraction from clinical microbiology text. We trained LLMs and ML models to extract specific and relevant antibiotic resistance information from free-text microbiology reports. Human curation of this data is currently necessary but time consuming because there are no interoperability standards in this domain,^4^ and EHRs often use free-text fields to convey key information,^5^ including antibiotic susceptibility testing (AST) results to last-line antibiotics and mechanisms of antibiotic resistance. Some laboratories are forced to rely on unstructured free-text fields rather than custom-built structured data elements due to limited laboratory information system technicians, low volumes for these specialized tests, and EHR differences. Improving electronic data standardization with LLM and ML technology in the infectious diseases domain will enable secondary uses, such as real-time outbreak detection, public health reporting, and developing and training AI/ML models that predict antibiotic resistance.

## Methods

### Study design

We used data from the VA Corporate Data Warehouse, which contains EHR data from all 136 VA Medical Centers. This dataset included microbiology comment boxes from bacterial culture reports from 10/1/99 to 2/11/22; each microorganism has one comment box with up to 8,000 characters. *A priori*, we chose to use iterative SQL queries to limit entries to under 10,000 due to limited annotation time (Supplemental Methods). The comment box is an unstructured free-text box used by microbiology laboratory technicians to communicate any important information that does not have a structured data field (Supplemental Figure 1), for example, results of susceptibility testing for antibiotics not in automated panels (*eg*, ceftazidime/avibactam, ceftolozane/tazobactam). The contents of the microbiology comment box and antibiotics tested on automated panels are not standardized across facilities.

### Model development and evaluation

We chose the ML models multinomial logistic regression, random forest, and XGBoost. Briefly, random forest generates multiple random decision trees, aggregates their results, and returns the most common prediction, whereas XGBoost sequentially generates decision trees where each tree attempts to correct errors by previous trees. Prior to training, the dataset was randomly split 80/20 for training/testing. The test set was not accessed until final evaluation. For ML models, Scikit-learn Pipeline function was used to link each step of training, including CountVectorizer, TfidfTransformer, and the ML model (Supplemental Figure 2). Pipeline hyperparameters were tuned using fivefold cross validation with GridSearchCV, and model hyperparameters with best F1_macro scores (unweighted macro-average across all groups) were used for final evaluation; F1 measure was selected as the primary performance measure due to the imbalanced class distribution.^6^ For LLM models, LLM tokenization was set to padding max_length (512) and truncation, and fine-tuned using the training dataset with Bayesian hyperparameter tuning optimized to minimize loss function. We chose Bayesian hyperparameter tuning because LLM grid search was computationally unfeasible and Bayesian approaches have been shown to perform similarly.^7^ See Supplemental Table 1 for hyperparameter tuning settings.

To evaluate whether LLM’s can be used out-of-the-box or whether expert-developed text-preprocessing rules (Supplemental Table 2) are necessary, we fine-tuned BioBERT^8^ using pre-processed text (BioBERT_preprocessed_) and trained BioBERT using raw text (BioBERT_raw_) for classification. To evaluate whether biomedical domain-specific models perform better, we fine-tuned BERT for classification and compared it to BioBERT models.

## Results

The final dataset contained 7,527 entries and were split into the training set with 6,021 entries and the test set with 1,506 entries; the test set was not accessed until final evaluation. 115 of 136 (84.5%) sites contributed at least one observation. Table 1 summarizes the algorithms’ performances during final evaluation on the test set (see Supplemental Figures 3–5 for confusion matrices). Error analysis identified misspellings, sound-alike antibiotics, and non-standard verbiage as common failure modes (Supplemental Results).

**Table 1.** Classification performance on the testing set across prediction tasks and algorithms

	F1^*^	Precision	Recall			
	score	(PPV)	(Sensitivity)	Specificity	NPV	Accuracy
Ceftazidime/avibactam AST classification						
Logistic regression	0.928	0.919	0.938	0.985	0.972	0.969
Random forest	0.765	0.950	0.726	0.950	0.726	0.945
XGBoost	0.951	0.972	0.934	0.987	0.984	0.980
BERT	0.976	0.977	0.975	0.993	0.991	0.989
BioBERT_preprocessed_	0.986	0.979	0.993	0.995	0.994	0.993
BioBERT_raw_	0.950	0.931	0.973	0.994	0.986	0.985
Ceftolozane/tazobactam AST classification						
Logistic regression	0.892	0.889	0.897	0.987	0.984	0.967
Random forest	0.816	0.956	0.748	0.982	0.988	0.965
XGBoost	0.934	0.986	0.894	0.990	0.992	0.979
BERT	0.796	0.892	0.718	0.985	0.989	0.966
BioBERT_preprocessed_	0.984	0.992	0.977	0.997	0.996	0.993
BioBERT_raw_	0.889	0.924	0.873	0.990	0.985	0.969
Carbapenemase status classification						
Logistic regression	0.931	0.929	0.933	0.981	0.984	0.989
Random forest	0.942	0.967	0.920	0.964	0.993	0.989
XGBoost	0.954	0.951	0.959	0.984	0.984	0.993
BERT	0.912	0.912	0.913	0.983	0.971	0.987
BioBERT_preprocessed_	0.907	0.943	0.876	0.960	0.995	0.987
BioBERT_raw_	0.924	0.924	0.926	0.991	0.977	0.989

Abbreviations: AST, antimicrobial susceptibility testing; PPV, positive predictive value; NPV, negative predictive value.

* F1 macro-averaging is reported (see Methods). F1 score is the measure of the harmonic mean of precision and recall.

### Classification for antimicrobial susceptibility test results

The performance of the ML and LLM algorithms varied by task (Table 1). For both antibiotic AST classification tasks, the ML algorithm with the best performance was XGBoost (F1 scores: ceftazidime/avibactam 0.951, ceftolozane/tazobactam 0.934), followed by logistic regression and random forest. For both antibiotic AST classification tasks, the LLM algorithm with the best performance was BioBERT_preprocessed_ (F1 scores: ceftazidime/avibactam 0.986, ceftolozane/tazobactam 0.984), and the rank order of BERT and BioBERT_raw_ varied by antibiotic.

Overall, the best-performing LLM performed better than the best ML model for both ceftazidime/avibactam classification and ceftolozane/tazobactam classification. The results also show performance gains using preprocessed text rather than raw text for BioBERT training, 0.986 vs. 0.950 (BioBERT_preprocessed_ vs BioBERT_raw_) for ceftazidime/avibactam classification, and 0.984 vs. 0.889 for ceftolozane/tazobactam classification (Table 1). Notably, all models performed with high specificity (range: 0.950–0.997).

### Classification for carbapenemase production testing

Among ML algorithms, XGBoost had the best F1 score of 0.954, for carbapenemase production testing classification, followed by random forest and logistic regression with F1 scores of 0.942 and 0.931, respectively. Among the LLMs, BioBERT_raw_ performed best and followed by BERT and BioBERT_preprocessed_ with F1 scores of 0.924 vs. 0.912 vs. 0.907, respectively (Table 1). For carbapenemase production classification, the best-performing ML model, XGBoost, performed better than the best-performing LLM, BioBERT_raw_ with F1 scores of 0.954 vs. 0.924, respectively. Notably, all models performed with high specificity (range: 0.960–0.991).

## Discussion

In this study, we trained and evaluated six LLM and ML models to extract three antibiotic-resistance concepts from free-text fields of microbiology reports, and we achieved excellent specificity (range: 0.950–0.997), sensitivity (range: 0.718–0.993), and F1 score (range: 0.765–0.986); F1 score is the measure of the harmonic mean of recall (also known as sensitivity) and precision (also known as positive predictive value). Our main finding is that the generally favorable performances, in particularly excellent specificity, demonstrate that these LLMs and ML models are candidate tools for these information extraction tasks.

This study has several strengths, including using AI/ML algorithms rather than rule-based algorithms for information extraction from free-text microbiology reports, evaluating models on unseen data (*ie*, the test set), and evaluating three information extraction tasks. This study builds upon prior works that developed rule-based algorithms that ingested free-text microbiology reports to classify whether bacteria grew in a culture,^4^ identify methicillin-resistant *Staphylococcus aureus* status,^5^ and alert facilities in real-time of patients admitted with MDROs.^6^

This study is subject to several limitations. First, the study evaluated the performance of algorithms’ classification rather than a real-world implementation; we view this step, validation of an algorithms’ performances on internal data, a prerequisite to deployment. Second, we chose BERT-based language models; it is possible that newer language models (*eg*, Me-LLaMA outperformed many prior open-source LLMs)^9^ may achieve better performance on our dataset. We also did not use VA GPT (Beta), a custom-developed LLM chatbot authorized for storage, processing, and transmission of both PII and PHI data behind the VA firewall. We also were unable to compare training costs. Third, our dataset size was limited due to limited annotation time; it is possible that more data will improve performance. Fourth, we did not compare LLM/ML approaches with alternatives, such as laboratory information system technicians creating structured data fields in each EHR.

In summary, we developed and validated three LLMs (BERT, BioBERT_preprocessed_, BioBERT_raw_) and three ML (logistic regression, random forest, XGBoost) models to ingest free-text microbiology reports and classify each report’s carbapenemase resistance status and antibiotic-resistance status to the clinically relevant last-line antibiotics ceftazidime/avibactam and ceftolozane/tazobactam. Our models demonstrated excellent specificity, and acceptable sensitivity and F1-scores, and can be considered a successful “test case” for LLM as an Augmented Intelligence^10^ system that increases efficiency of manual chart review. Our findings support further work for more complex chart review activities using state-of-the-art models that are entering healthcare and potential for pilot deployment in public health surveillance, coordination between different hospital networks, and research model development.

## Supporting information

Chou et al. supplementary material 1 Chou et al. supplementary material

Chou et al. supplementary material 2 Chou et al. supplementary material

## Data Availability

Study data are available through the corresponding author in accordance with VHA Handbook 1200.12 and the local IRB.

## Supplementary material

The supplementary material for this article can be found at https://doi.org/10.1017/ice.2025.10210

## Manuscript preparation

LLMs were only used as described in the study methods; LLMs were not used in any aspect of the preparation of the manuscript text.
