# Supplementary material for: Extracting antibiotic susceptibility from free-text microbiology reports using natural language processing
Source: Infect Control Hosp Epidemiol. 2025 Jul 31;46(9):941–3. doi: 10.1017/ice.2025.10210 (PMC12616221; doi:10.1017/ice.2025.10210)
Supplement: Chou et al. supplementary material 1 — Chou et al. supplementary material [file S0899823X25102109sup001.docx]

Supplemental Materials

Table of Contents

[**Methods**: Labelling 2](#_Toc195009211)

[**Methods**: Model Development and Evaluation 2](#_Toc195009212)

[**Results**: Error analysis 3](#_Toc195009213)

[**Supplemental Figure 1**: Sample microbiology free-text field 4](#_Toc195009214)

[**Supplemental Figure 2**: Hyperparameter Tuning Using GridSearchCV and Pipeline 5](#_Toc195009215)

[**Supplemental Figure 3**: Ceftazidime/Avibactam Antibiotic Susceptibility Testing Classification 6](#_Toc195009216)

[**Supplemental Figure 4**: Ceftolozane/Tazobactam Antibiotic Susceptibility Testing Classification 7](#_Toc195009217)

[**Supplemental Figure 5**: Carbapenemase Status Classification 8](#_Toc195009218)

[**Supplemental Table 1**: Hyperparameter Tuning Grids and Parameters 9](#_Toc195009219)

[**Supplemental Table 2**: Text Preprocessing: 10](#_Toc195009220)

[**Supplemental Table 3**: CDW Query: 11](#_Toc195009221)

[**Supplemental Table 4**: Classification performance on the testing set across prediction tasks and algorithms, excluding “test not done” class 12](#_Toc195009222)

# **Methods**: Labelling

Each microbiology report was manually reviewed by an infectious diseases specialist (AC), and assigned three independent categorical labels: ceftazidime/avibactam, ceftolozane/tazobactam, and carbapenemase-producer. The antibiotics, ceftazidime/avibactam and ceftolozane/tazobactam, were labelled as Susceptible (S), Intermediate (I), Resistant (R), or Not Reported (NR). The carbapenemase-producer status was labelled as Carbapenemase-Producer (CP), Non-Carbapenemase-Producer (NCP), or Not Reported (NR). Non-carbapenemase producers are where the isolate underwent test(s) for carbapenemase(s) and all carbapenemase tests were negative or not detected.

We chose ceftazidime/avibactam and ceftolozane/tazobactam AST results for LLM and ML classification because they were likely to be add-on tests that appears in the microbiology comment fields and they are among the most clinically important therapies for KPC-producing Enterobacterales and difficult-to-treat *Pseudomonas aeruginosa* (Tamma 2024).

*A priori*, we chose to limit the size of the dataset to under 10,000 entries due to feasibility of manually reviewing and labeling each entry. We iteratively queried the CDW microbiology comment fields for last-line antibiotics using generic names, trade names, and with wildcards (Supplemental Table 3). Data from Cerner Millennium EHR sites were excluded due to lack of data standardization and unique microbiology schema in CDW.

We chose ceftazidime/avibactam, ceftolozane/tazobactam, and carbapenemase tests because these were the earliest FDA-approved novel antibiotics, and because there is only partial overlap in their spectrums of activity (IDSA AMR Guidance), which is expected to generate a more diverse corpus (*i.e.*, reports can contain only ceftazidime/avibactam result, only ceftolozane/tazobactam result, both results, or neither result).

# **Methods**: Model Development and Evaluation

We developed ML and LLM models to extract ceftazidime/avibactam status, ceftolozane/tazobactam status, and carbapenemase status from each free-text microbiology entry. Models were trained using NVIDIA Tesla V100 Tensor Core GPUs and NVIDIA T4 Tensor Core GPUs on the VA Research Analytics Science Platform (RASP), a secure deployment of Amazon Web Services GovCloud within the VA Enterprise Cloud (VAEC-AWS).

Software packages used were Python 3.10, scikit-learn 1.2, XGBoost 2.1.1, PyTorch 1.13.1, Transformers 4.26.0, BERT-base-cased, BioBERT-base-cased v1.1. BERT, is a language representation model containing 110 million parameters, and BioBERT is a BERT-based domain-specific model pre-trained on large-scale biomedical corpora (Lee 2020).

Multinomial logistic regression was used for multiclass classification (*i.e.*, more than >3 predicted outcomes). Outputs were: predicted class/outcomes which were used in confusion matrixes, and predicted probability of each class via the softmax function (for additional details, see: <https://scikit-learn.org/stable/modules/generated/sklearn.linear_model.LogisticRegression.html#sklearn.linear_model.LogisticRegression>) during error analysis.

# **Results**: Error analysis

To gain further insight into the performance of these models, we reviewed the error from them. We identified different patterns for antibiotic susceptibility result classification (*i.e.*, ceftazidime/avibactam and ceftolozane/tazobactam) and resistance mechanism result classification (*i.e.*, carbapenemase testing).

In error analysis of carbapenemase testing results, the most common errors were due to inability to recognize carbapenemase groups, as opposed to individual carbapenemases, and not recognizing results using non-standard verbiage. For example, a carbapenemase group term, such as "metallo-beta-lactamase positive", would be incorrectly classified, while a specific carbapenemase in the metallo-beta-lactamase group term, such as "NDM not detected" would be correctly classified. Carbapenemase result classification errors also were due to non-standard result verbiage, such as "demonstrated" and "confirmed", while verbiage consistent with the manufacturers’ documentation (*e.g.*, "detected", "not detected", "no result") were correctly classified.

Error analyses of antibiotic susceptibility result classifications were similar for ceftazidime/avibactam and ceftolozane/tazobactam. The most common errors were due to misspellings (*e.g.*, "sensitve", "ceftotaxime") and sound-alike antibiotics. Sound-alike antibiotics led to errors, such as failure to differentiate ceftazidime from ceftazidime/avibactam, and ceftizoxime from ceftazidime/avibactam; these antibiotics (*e.g.*, ceftizoxime , ceftazidime) were rarely reported in the free-text fields and usually reported as structured data elements. We considered correcting misspellings during preprocessing but felt it could lead to over-fitting.

# **Supplemental Figure 1**: Sample microbiology free-text field

DD/MM/YY Possible carbapenem resistant Enterobacteriaceae (CRE) present. Further testing in progress. Contact infection control for guidance. DD/MM/YY:Carbapenemase PCR results are negative.No CRE isolated. NDM:NOT DETECTED KPC:NOT DETECTED Ceftazidime-Avibactam susceptible Ceftolozane/Tazobactam MIC= 0.5 susceptible Vabomere (meropenem/vaborbactam) susceptible

Sample text found in microbiology free-text fields. Each color shows a concept, including the test name and result.

# **Supplemental Figure 2**: Hyperparameter Tuning Using GridSearchCV and Pipeline

GridSearchCV was executed for each task (ceftazidime/avibactam, ceftolozane/tazobactam, carbapenemase) and ML classifier (XGBoost, random forest, logistic regression) combination; there were a total of 9 combinations.

Note: see separate supplemental PDF file for enlarged version.

| **Supplemental Figure 3**: Ceftazidime/Avibactam Antibiotic Susceptibility Testing Classification | | |
| --- | --- | --- |
| a. Logistic regression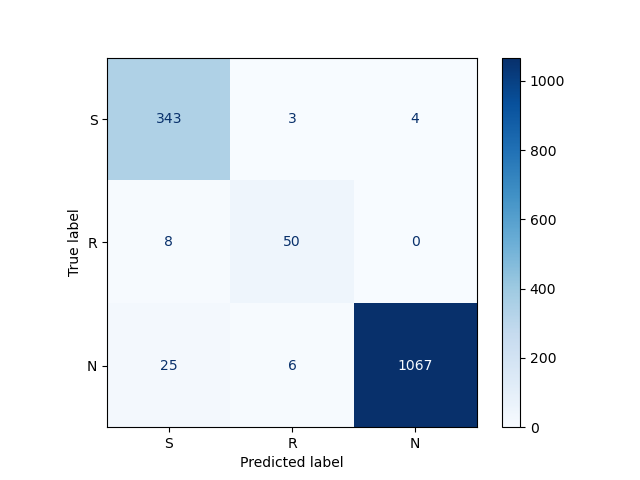 | b. Random forest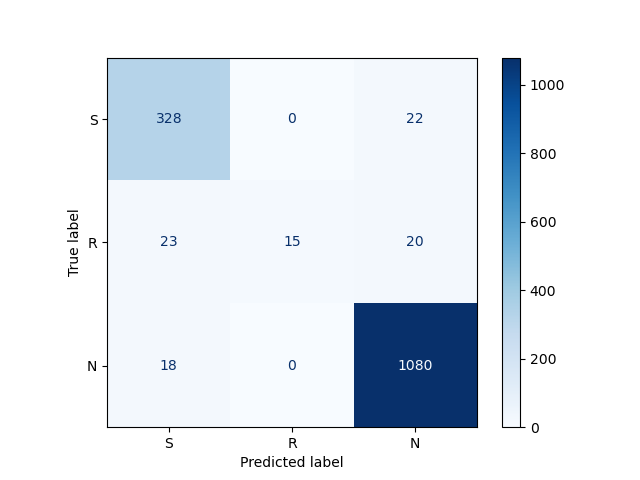 | c. XGBoost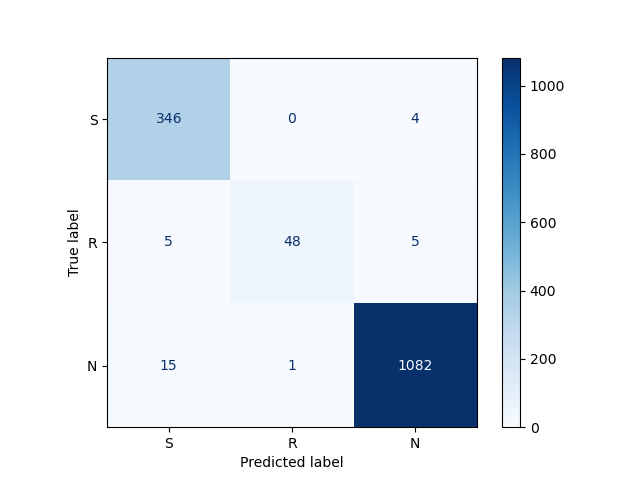 |
| d. BERT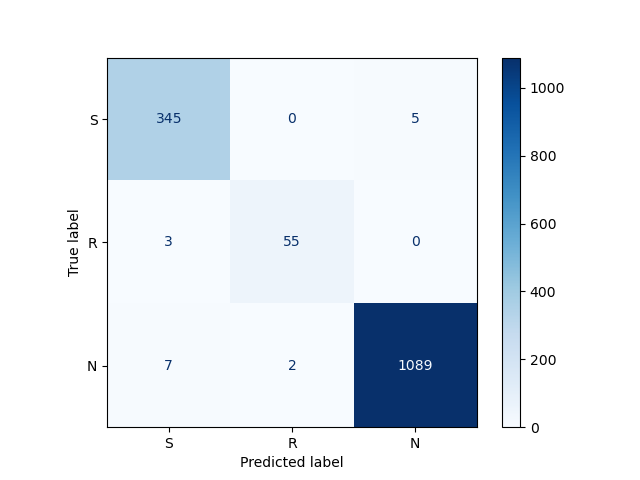 | e. BioBERT_preprocessed_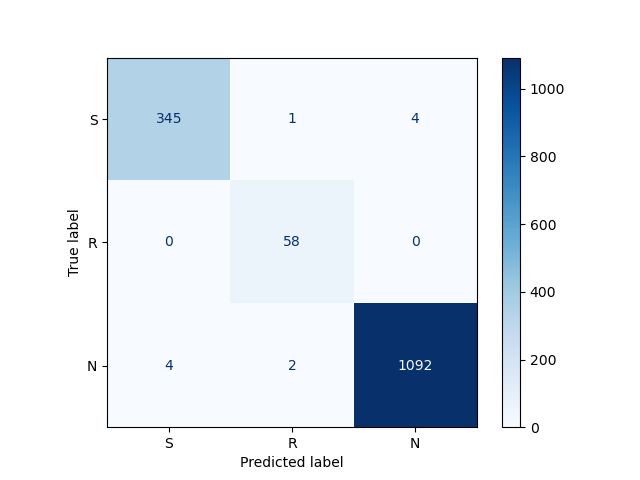 | f. BioBERT_raw_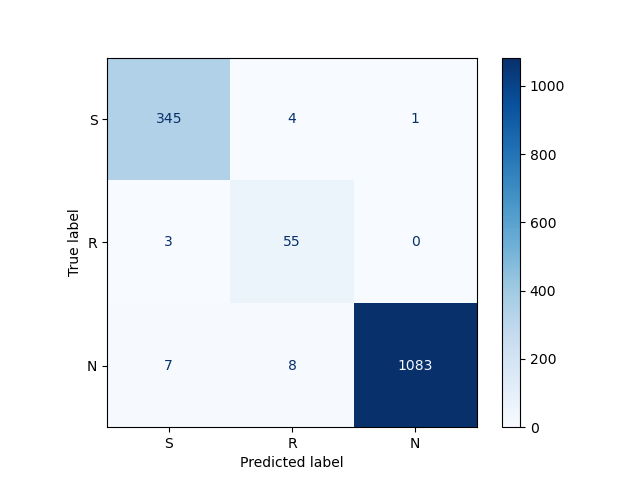 |

The frequency of true label were: Susceptible 350/1506 (23.2%), Resistant 58/1506 (3.9%), Not tested 1098/1506 (72.9%).

S: Susceptible.

R: Resistant.

N: Not tested.

| **Supplemental Figure 4**: Ceftolozane/Tazobactam Antibiotic Susceptibility Testing Classification | | |
| --- | --- | --- |
| a. Logistic regression  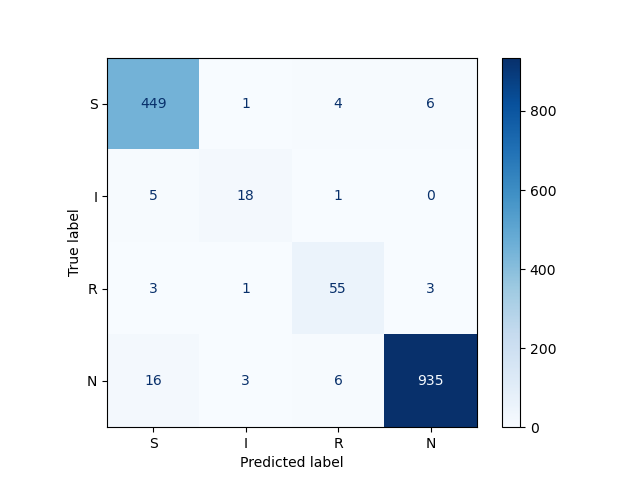 | b. Random forest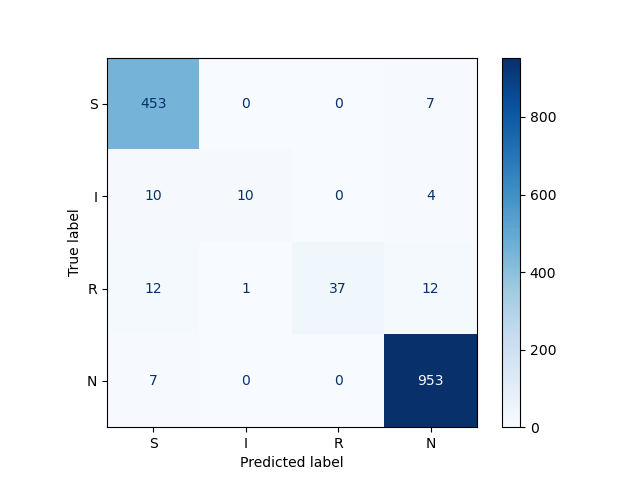 | c. XGBoost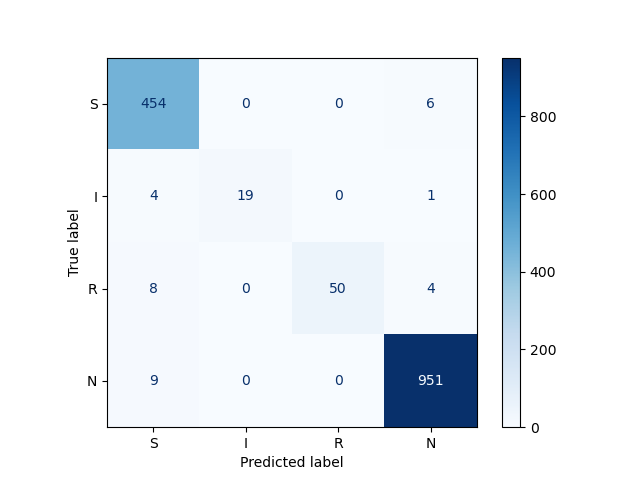 |
| d. BERT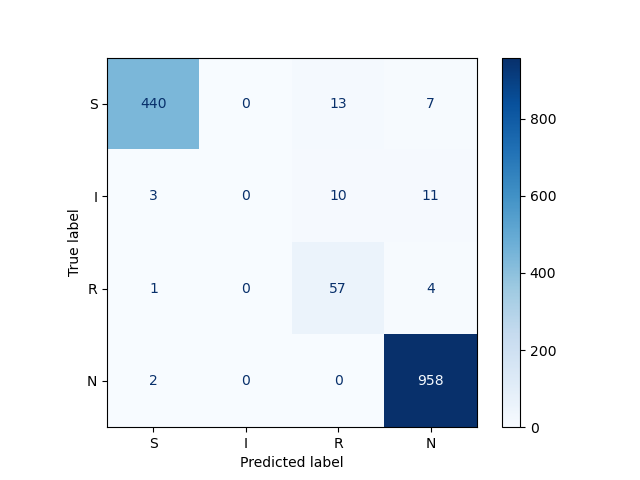 | e. BioBERT_preprocessed_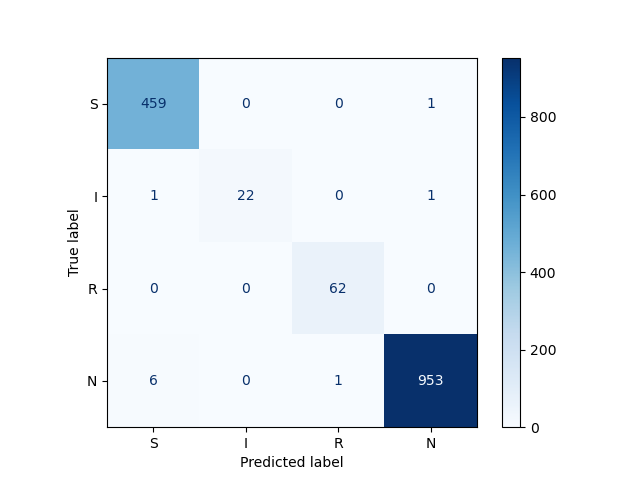 | f. BioBERT_raw_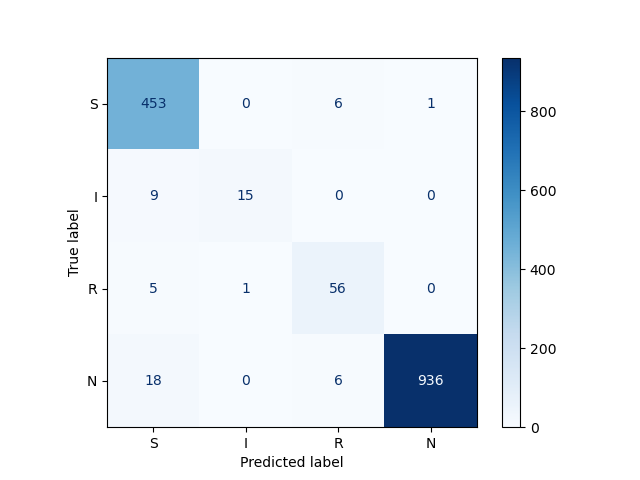 |

The frequency of true label were: Susceptible 460/1506 (30.5%), Intermediate 24/1506 (1.6%), Resistant 62/1506 (4.1%), Not tested 960/1506 (63.7%).

S: Susceptible.

I: Intermediate

R: Resistant.

N: Not tested.

| **Supplemental Figure 5**: Carbapenemase Status Classification | | |
| --- | --- | --- |
| a. Logistic regression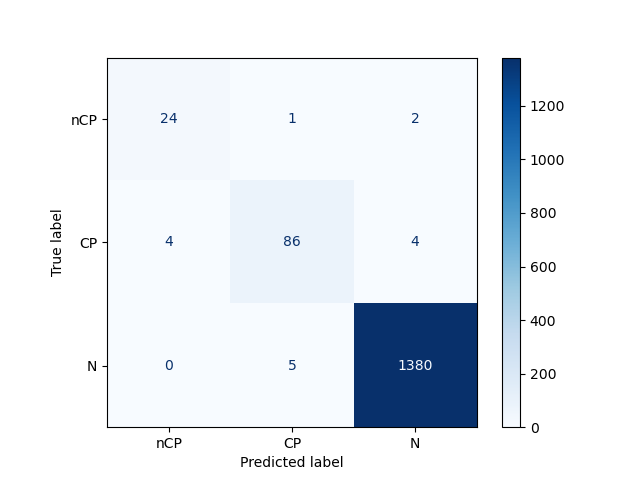 | b. Random forest 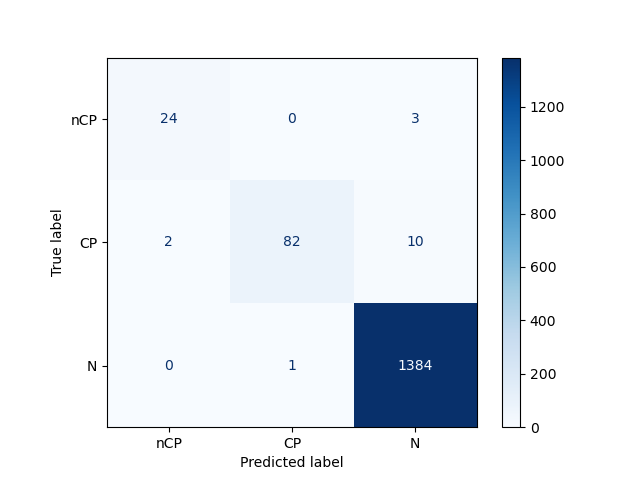 | c. XGBoost 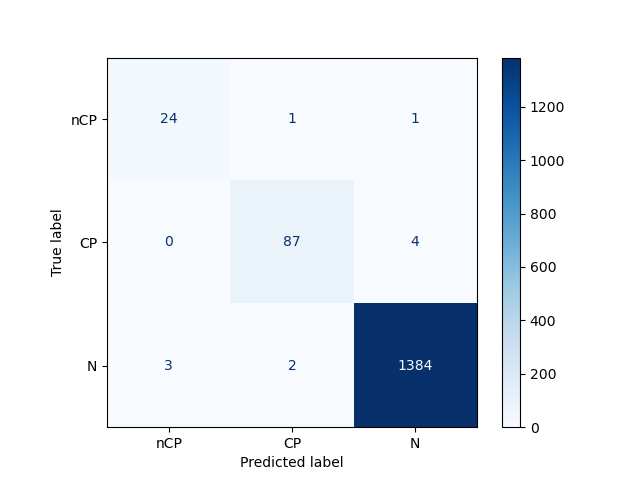 |
| d. BERT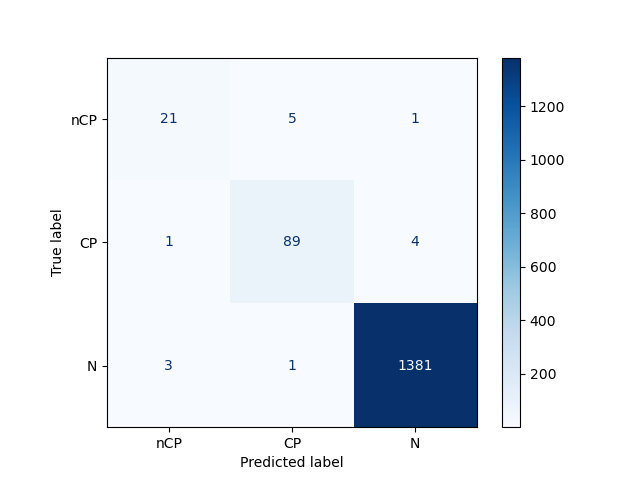 | e. BioBERT_preprocessed_ 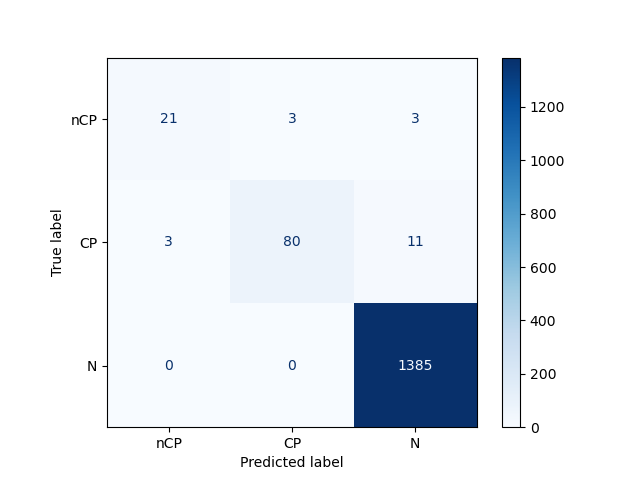 | f. BioBERT_raw_ 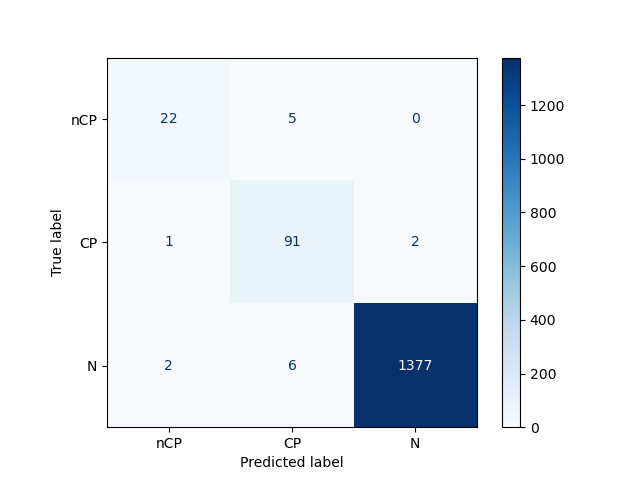 |

The frequency of true label were: non-carbapenemase producer 26/1506 (1.7%), carbapenemase producer 91/1506 (6.0%), Not tested 1389/1506 (92.2%).

nCP: non-carbapenemase producer.

CP: carbapenemase producer.

N: not tested.

# **Supplemental Table 1**: Hyperparameter Tuning Grids and Parameters

| Model | Hyperparameter grid | Best parameters,  Ceftazidime/avibactam | Best parameters,  Ceftolozane/tazobactam | Best parameters,  Carbapenemase |
| --- | --- | --- | --- | --- |
| XGBoost | 'vect__ngram_range': [(1,1), (1,2),(1,4),(1,8)],  'tfidf__use_idf': [True, False],  'clf-xgb__min_child_weight': [1, 5, 10],  'clf-xgb__gamma': [0.5, 1, 1.5, 2, 5],  'clf-xgb__subsample': [0.6, 0.8, 1.0],  'clf-xgb__colsample_bytree': [0.6, 0.8, 1.0],  'clf-xgb__max_depth': [3, 4, 5] | 'clf-xgb__colsample_bytree': 1.0,  'clf-xgb__gamma': 0.5,  'clf-xgb__learning_rate': 0.1,  'clf-xgb__max_depth': 5,  'clf-xgb__min_child_weight': 1,  'clf-xgb__subsample': 1.0,  'tfidf__use_idf': True,  'vect__ngram_range': (1, 4) | 'clf-xgb__colsample_bytree': 0.6,  'clf-xgb__gamma': 1,  'clf-xgb__learning_rate': 0.01,  'clf-xgb__max_depth': 7,  'clf-xgb__min_child_weight': 1,  'clf-xgb__subsample': 1.0,  'tfidf__use_idf': False,  'vect__ngram_range': (1, 8) | 'clf-xgb__colsample_bytree': 1.0,  'clf-xgb__gamma': 1,  'clf-xgb__learning_rate': 0.1,  'clf-xgb__max_depth': 3,  'clf-xgb__min_child_weight': 1,  'clf-xgb__subsample': 1.0,  'tfidf__use_idf': True,  'vect__ngram_range': (1, 4) |
| Random Forest | 'vect__ngram_range': [(1,1), (1,2),(1,4),(1,8)],  'tfidf__use_idf': [True, False],  'clf-rf__criterion': ['gini', 'entropy'],  'clf-rf__max_depth': [1,2,4,8,16,32],  'clf-rf__n_estimators': [10,100,1000],  'clf-rf__min_samples_leaf': [0.01, 0.1, 0.4, 1],  'clf-rf__min_samples_split': [0.01, 0.1, 0.2, 0.4] | 'clf-rf__criterion': 'entropy',  'clf-rf__max_depth': 32,  'clf-rf__min_samples_leaf': 1,  'clf-rf__min_samples_split': 0.01,  'clf-rf__n_estimators': 10,  'tfidf__use_idf': False,  'vect__ngram_range': (1, 4) | 'clf-rf__criterion': 'entropy',  'clf-rf__max_depth': 32,  'clf-rf__n_estimators': 10,  'clf-rf__min_samples_leaf': 1,  'clf-rf__min_samples_split': 0.01,  'tfidf__use_idf': True,  'vect__ngram_range': (1,2) | 'clf-rf__criterion': 'gini',  'clf-rf__max_depth': 32,  'clf-rf__min_samples_leaf': 1,  'clf-rf__min_samples_split': 0.01,  'clf-rf__n_estimators': 100,  'tfidf__use_idf': True,  'vect__ngram_range': (1, 2) |
| Logistic regression | 'vect__ngram_range': [(1,1),(1,2),(1,4),(1,8)],  'tfidf__use_idf': [True, False],  'clf-lr__C': [100, 10, 1.0, 0.1, 0.01],  'clf-rf__penalty': ['l1', 'l2', 'elasticnet'],  'clf-rf__solver': ['lbfgs', 'liblinear', 'newton-cg', 'sag', 'saga'],  'clf-rf__class_weight': ['balanced', None]," | 'clf-lr__C': 10,  'clf-lr__class_weight': 'balanced',  'clf-lr__penalty': 'l1',  'clf-lr__solver': 'liblinear',  'tfidf__use_idf': True,  'vect__ngram_range': (1, 2) | 'clf-lr__C': 10,  'clf-lr__class_weight': 'balanced',  'clf-lr__penalty': 'l1',  'clf-lr__solver': 'liblinear',  'tfidf__use_idf': True,  'vect__ngram_range': (1, 4) | 'clf-lr__C': 100,  'clf-lr__class_weight': 'none',  'clf-lr__penalty': 'l1',  'clf-lr__solver': 'liblinear',  'tfidf__use_idf': True,  'vect__ngram_range': (1, 2) |

# **Supplemental Table 2**: Text Preprocessing

| Preprocessing step | Regular Expressions |
| --- | --- |
| Retained pre-specified characters (>=, =, <=) | r'>=', ' greaterthan '  r'<=', ' lessthan '  r'=', ' equals ' |
| Tokenize punctuation | r’([><!@#%\^&\*\(\):;"\',\./\\-]+)', r' \1 ', |
| Remove time of day | r'[0-9]+\s*(am\|pm\|AM\|PM)($\|\s)' |
| Remove numbers | r'[0-9]+' |
| Expanded abbreviations | r'\b[Rr]($\|\s)', ' resistant '  r'\b[Ii]($\|\s)', ' intermediate '  r'\b[Ss]($\|\s)', ' susceptible '  r'\bC[\s]+[/-][\s]+A($\|\s)', ' ceftazidime avibactam '  r'\bC[\s]+[/-][\s]+T($\|\s)', ' ceftolozane tazobactam ' |
| Uncase | str.lower() |
| Expand unambiguous abbreviated antibiotics | r'\bceftaz[a-z]*($\|\s)', ' ceftazidime '  r'\bav[iy][a-z]*($\|\s)', ' avibactam '  r'\bcefto[a-z]*($\|\s)', ' ceftolozane '  r'\btaz[a-z]*($\|\s)', ' tazobactam '  r'\bpip[a-z]*($\|\s)', ' piperacillin ' |
| Replace trade names with generic names | r'\bav[iy][a-z]*z($\|\s)', ' ceftazidime avibactam '  r'\b(z[a-z]*xa)($\|\s)', ' ceftolozane tazobactam '  r'\b(zos[a-z]*)($\|\s)', ' piperacillin tazobactam ' |
| Change sensitive to susceptible | r'\bsensitive($\|\s)', ' susceptible ' |
| Change detected to positive | r'\bnot\s+detected($\|\s)', ' negative '  r'\bdetected($\|\s)', ' positive ' |
| Converted gene names to encoded proteins | r'\b(blakpc)($\|\s)', ' kpc '  r'\b(blandm)($\|\s)', ' ndm '  r'\b(blaoxa)($\|\s)', ' oxa '  r'\b(blavim)($\|\s)', ' vim '  r'\b(blaimp)($\|\s)', ' imp '  r'\b(bla)($\|\s)', ' ' |
| Remove measurement units | r'\b(ug)($\|\s)', ' '  r'\b(mcg)($\|\s)', ' '  r'\b(ml)($\|\s)', ' '  r'\b(mg)($\|\s)', ' '  r'\b(mm)($\|\s)', ' ' |
| Remove susceptibility test assay types | r'\b(mic)($\|\s)', ' '  r'\b(kirby)($\|\s)', ' '  r'\b(bauer)($\|\s)', ' '  r'\be\s*test($\|\s)', ' ' |

# **Supplemental Table 3**: CDW Query

| **Search String** | **Generic (capitalized) or generic (lower-case) names** |
| --- | --- |
| ‘%Avy%’ | Avycaz (ceftazidime/avibactam) |
| ‘%Avib%’ | avibactam |
| ‘%Zerb%’ | Zerbaxa (ceftolozane/tazobactam) |
| ‘%cefto%’ | ceftolozane |
| ‘%Vabom%’ | Vabomere (meropenem/vaborbactam) |
| ‘%vabor%’ | vaborbactam |
| ‘%Recar%’ | Recarbrio (Imipenem/cilastatin/relebactam) |
| ‘%releb%’ | relebactam |

# **Supplemental Table 4**: Classification performance on the testing set across prediction tasks and algorithms, excluding “test not done” class

|  |  | **F1* score** | **Precision (PPV)** | **Recall (Sensitivity)** | **Specificity** | **NPV** | **Accuracy** |
| --- | --- | --- | --- | --- | --- | --- | --- |
| **Ceftazidime/avibactam AST classification** | | | | | | | |
|  | Logistic regression | 0.940 | 0.960 | 0.921 | 0.927 | 0.927 | 0.968 |
|  | Random forest | 0.673 | 0.967 | 0.598 | 0.802 | 0.752 | 0.892 |
|  | XGBoost | 0.946 | 0.993 | 0.908 | 0.957 | 0.951 | 0.977 |
|  | BERT | 0.981 | 0.996 | 0.967 | 0.974 | 0.954 | 0.987 |
|  | BioBERT_preprocessed_ | 0.992 | 0.992 | 0.993 | 0.999 | 0.960 | 0.993 |
|  | BioBERT_raw_ | 0.964 | 0.962 | 0.967 | 0.968 | 0.954 | 0.982 |
| **Ceftolozane/tazobactam AST classification** | | | | |  |  |  |
|  | Logistic regression | 0.900 | 0.933 | 0.871 | 0.964 | 0.950 | 0.976 |
|  | Random forest | 0.763 | 0.954 | 0.666 | 0.914 | 0.942 | 0.958 |
|  | XGBoost | 0.916 | 0.985 | 0.862 | 0.926 | 0.964 | 0.974 |
|  | BERT | 0.888 | 0.852 | 0.625 | 0.969 | 0.916 | 0.954 |
|  | BioBERT_preprocessed_ | 0.985 | 0.999 | 0.971 | 0.996 | 0.995 | 0.998 |
|  | BioBERT_raw_ | 0.877 | 0.937 | 0.838 | 0.941 | 0.965 | 0.974 |
| **Carbapenemase status classification** | | | |  |  |  |  |
|  | Logistic regression | 0.912 | 0.923 | 0.902 | 0.960 | 0.866 | 0.934 |
|  | Random forest | 0.919 | 0.962 | 0.881 | 0.989 | 0.830 | 0.930 |
|  | XGBoost | 0.966 | 0.994 | 0.940 | 0.981 | 0.920 | 0.970 |
|  | BERT | 0.913 | 0.961 | 0.873 | 0.939 | 0.914 | 0.946 |
|  | BioBERT_preprocessed_ | 0.864 | 0.919 | 0.814 | 0.928 | 0.785 | 0.893 |
|  | BioBERT_raw_ | 0.919 | 0.952 | 0.891 | 0.902 | 0.914 | 0.942 |

Metrics computed excluding entries labelled as “test not done”.

Abbreviations: AST, antimicrobial susceptibility testing; PPV, positive predictive value; NPV, negative predictive value.

* F1 macro-averaging is reported (see Methods). F1 score is the measure of the harmonic mean of precision and recall.
