# Supplementary figures and images for: Extracting antibiotic susceptibility from free-text microbiology reports using natural language processing
Source: Infect Control Hosp Epidemiol. 2025 Jul 31;46(9):941–3. doi: 10.1017/ice.2025.10210 (PMC12616221; doi:10.1017/ice.2025.10210)

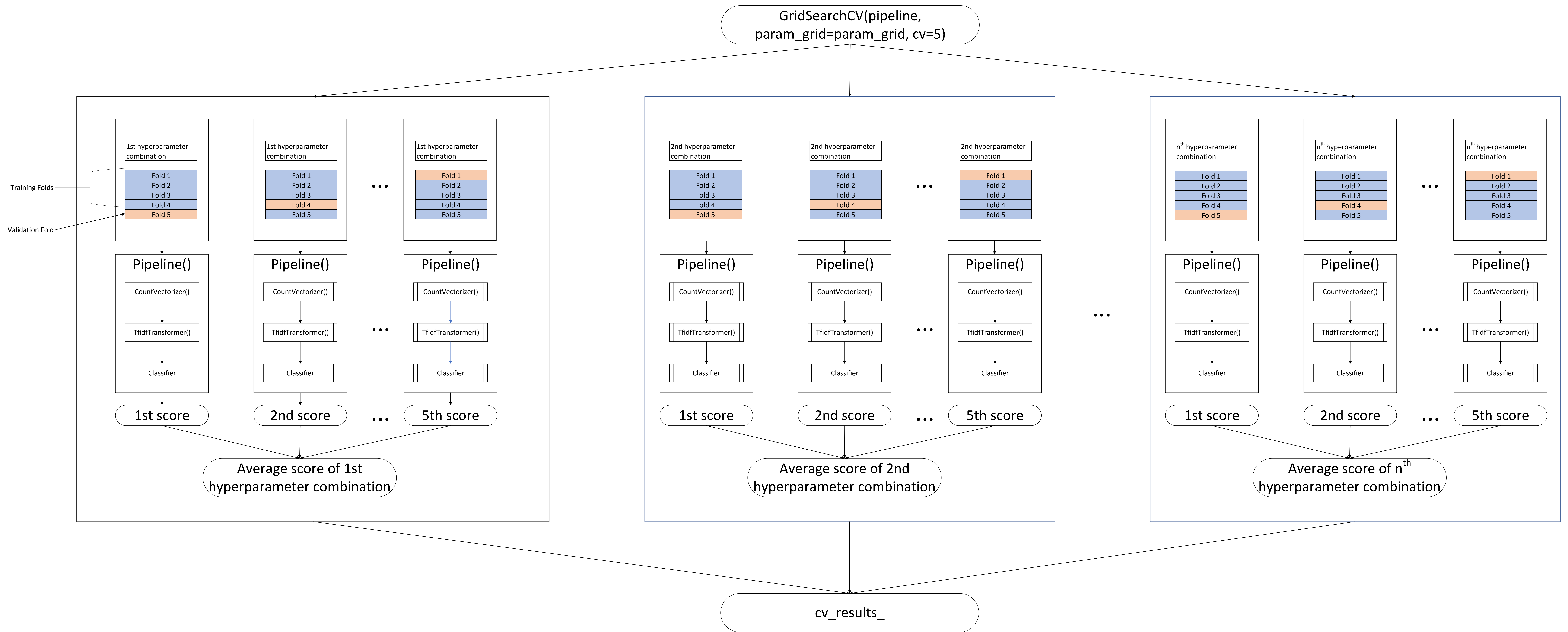

Supplement: Chou et al. supplementary material 2 — Chou et al. supplementary material [file S0899823X25102109sup002.pdf]
